# The tumor suppressor p53 can promote collective cellular migration

**DOI:** 10.1371/journal.pone.0202065

**Published:** 2019-02-01

**Authors:** Shijie He, Christopher V. Carman, Jung Hyun Lee, Bo Lan, Stephan Koehler, Lior Atia, Chan Young Park, Jae Hun Kim, Jennifer A. Mitchel, Jin-Ah Park, James P. Butler, Quan Lu, Jeffrey J. Fredberg

**Affiliations:** 1 Harvard T.H. Chan School of Public Health, Boston, Massachusetts, United states of America; 2 Massachusetts General Hospital and Harvard Medical School, Charlestown, Massachusetts, United states of America; Pennsylvania State Hershey College of Medicine, UNITED STATES

## Abstract

Loss of function of the tumor suppressor p53 is known to increase the rate of migration of cells transiting the narrow pores of the traditional Boyden chamber assay. Here by contrast we investigate how p53 impacts the rate of cellular migration within a 2D confluent cell layer and a 3D collagen-embedded multicellular spheroid. We use two human carcinoma cell lines, the bladder carcinoma EJ and the colorectal carcinoma HCT116. In the confluent 2-D cell layer, for both EJ and HCT cells the migratory speeds and effective diffusion coefficients for the p53 null cells were significantly smaller than in p53-expressing cells. Compared to p53 expressers, p53-null cells exhibited more organized cortical actin rings together with reduced front-rear cell polarity. Furthermore, loss of p53 caused cells to exert smaller traction forces upon their substrates, and reduced formation of cryptic lamellipodia. In the 3D multicellular spheroid, loss of p53 consistently reduced collective cellular migration into surrounding collagen matrix. As regards the role of p53 in cellular migration, extrapolation from the Boyden chamber assay to other cellular microenvironments is seen to be fraught even in terms of the sign of the effect. Together, these paradoxical results show that the effects of p53 on cellular migration are context-dependent.

## Introduction

Among human cancers, the tumor suppressor p53 is the most mutated gene and serves not only as an inducer of cancer cell senescence and apoptosis [1,2], but also as a central suppressor of cancer cell migration and metastasis [3–6]. In 3-dimensional (3D) Matrigel assays, for example, loss of p53 increases single cell invasion by enhancing cell contractility [7–10]. In 2D scratch wound healing assays, p53 can decrease the migration distance of leading cells by the inhibition of epithelial-mesenchymal transition (EMT) [11]. In addition, p53 can inhibit cancer cell metastasis by suppressing focal adhesion kinase (FAK) [12] and preventing degradation of the extracellular cell matrix (ECM) [3,13].

As regards the effects of p53 on cell migration, studies to date have emphasized measurements using the Matrigel-coated Boyden chamber assay [7–10]. The Boyden chamber assay measures the rate of transit of cells through narrow pores, typically 8 μm in diameter, wherein opportunities for cell-cell contact and resulting collective and cooperative cellular interactions are possible but, as a result of the geometry, are highly constrained. It is now recognized, however, that cell migration in metastatic disease is mainly collective [14–16], wherein cell-cell interactions can be strong and cooperative [17–21]. Moreover, the cellular collective can become jammed, immobile, and solid-like, or unjammed, mobile, and fluid-like [18,22–25]. It remains unclear, however, how p53 functions in the context of such collective phenomena.

To address that issue, here we studied migration in the 2D confluent cell layer and the 3D collagen-embedded multicellular spheroid. Two human cell lines were used, the bladder carcinoma EJ and the colorectal carcinoma HCT116. We first replicated single cell migration assays in the Boyden chamber and found results consistent with previous studies [7–9]; loss of p53 increased the migration of the single carcinoma cell. To our surprise, however, loss of p53 in either EJ or HCT 116 cells suppressed cellular diffusion in 2-D confluent cell layers. In that case, loss of p53 was also associated with reduced lamellipodia formation and weaker cell-substrate interactions. To better mimic tumor biology we also conducted studies using 3D multicellular spheroids embedded in collagen matrix. We found these results in the 3D multicellular spheroidal assay to be consistent with the 2D confluent assay. These results, taken together, demonstrate that the effects of p53 on cellular migration are context-dependent and sometimes paradoxical.

## Results

Study design emphasizes pairwise comparisons (presence or absence of functional p53) within each of three different migration assays.

### In 2D confluent cell layers, p53 increases collective cellular motility

To determine the function of p53 in collective cell motility, we used both gain and loss of p53 function in colorectal and bladder carcinoma cell lines: stable wild type (p53^+/+^) and stable p53 null (p53^-/-^) HCT 116 and Tet-off inducible EJ cell line (Methods). In EJ cell line, p53 knock out (EJ p53 off) was established by the addition of doxycycline to the culture media. We began by replicating assays of single cell invasion in the Matrigel-coated Boyden chamber as reported in previous studies [7–10], wherein cells transit pores of 8 μm diameter under the influence of a chemoattractant (S1 Fig). During pore transit, cells in the apical chamber (S2 Fig) migrate to the basal chamber. We found consistent results in which loss of p53 increased cell migration (125±53 versus 415±101 cells per well, p = 0.009 for EJ; 249±65 versus 891±239, p = 0.03 for HCT 116) (S1 Fig).

**Fig 1:**
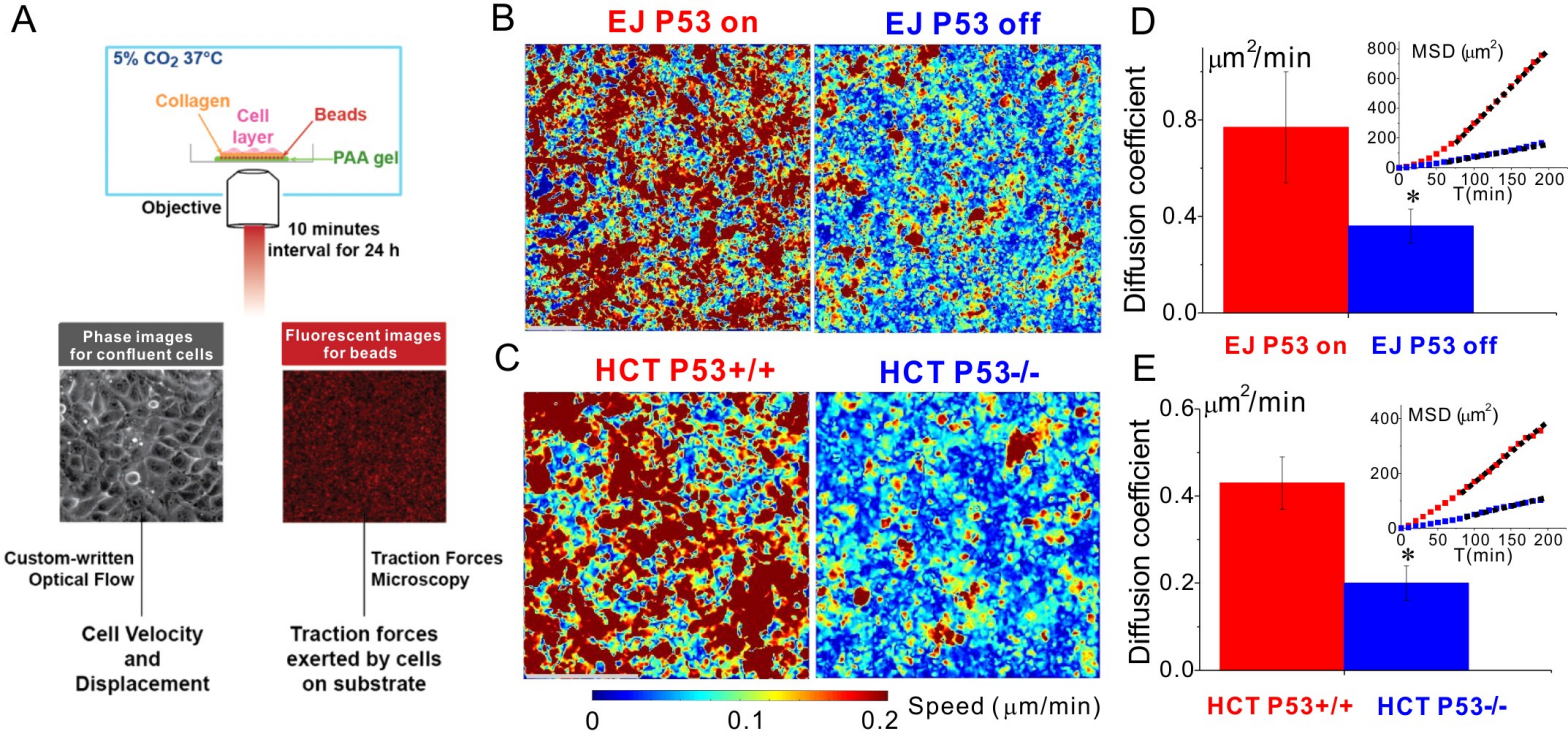
Loss of p53 reduces carcinoma motility and diffusion in confluent 2-D assay system. **A.** Cell velocity and displacement in the confluent cell layer were calculated based on the cell phase images by using custom-written optical flow. Traction forces exerted by the confluent cell layer on the Polyacrylamide (PAA) gel were derived from the florescent images of beads by using traction force microscopy (Method). (**B** and **C**) Speed maps show that loss of p53 decreases the speed of EJ cells and HCT116 cells. During 24h, their mean speeds are 0.12±0.003 versus 0.16±0.008 μm min^-1^, p = 0.002 for EJ; 0.11±0.018 versus 0.18±0.025 μm min^-1^, p = 0.0003 for HCT 116. (**D** and **E**) The diffusion coefficients, D, of EJ p53 off cells (HCT 116 p53^-/-^ cells) were smaller than EJ p53 on cells (HCT 116 p53^+/+^ cells). As such, p53 null cells were less diffusive than p53 expressing counterparts. D was calculated by linear fitting the mean squared displacements, MSDs (insets). Sample number n = 6~8, * represents p<0.05, Scale bar, 100 μm.

To study cellular migration in the context of the 2D confluent cell layer, we cultured cells on a 1.2 kPa polyacrylamide gel in which red fluorescent beads were embedded just below the gel surface. Using the Leica DMI8, we acquired phase images of the cell layer and fluorescent images of the red beads at 10 minutes interval for 24 h. Based on the phase images we calculated cell velocities and displacements using optical flow [26] (Methods and Fig 1A).

To our surprise, mean cell speeds for the p53 null version (EJ p53 off and HCT116 p53^-/-^) were smaller compared to their p53 expressing counterparts (0.12±0.003 versus 0.16±0.01 μm min^-1^, p = 0.002 for EJ; 0.11±0.02 versus 0.18±0.02 μmmin^-1^, p = 0.0003 for HCT 116; Fig 1B and 1C). When cell motions were expressed as an effective diffusion coefficient, the diffusivities of p53 null cells were consistently smaller than those of p53 expressing controls (0.36±0.07 versus 0.77±0.23 μm^2^ min^-1^, p = 0.003 for EJ; 0.20±0.05 versus 0.43±0.06 μm^2^ min^-1^, p = 0.0003 for HCT 116 in Fig 1D and 1E and S1 and S2 Movies).

### P53 null cells show weaker cell-substrate interactions

**Fig 2:**
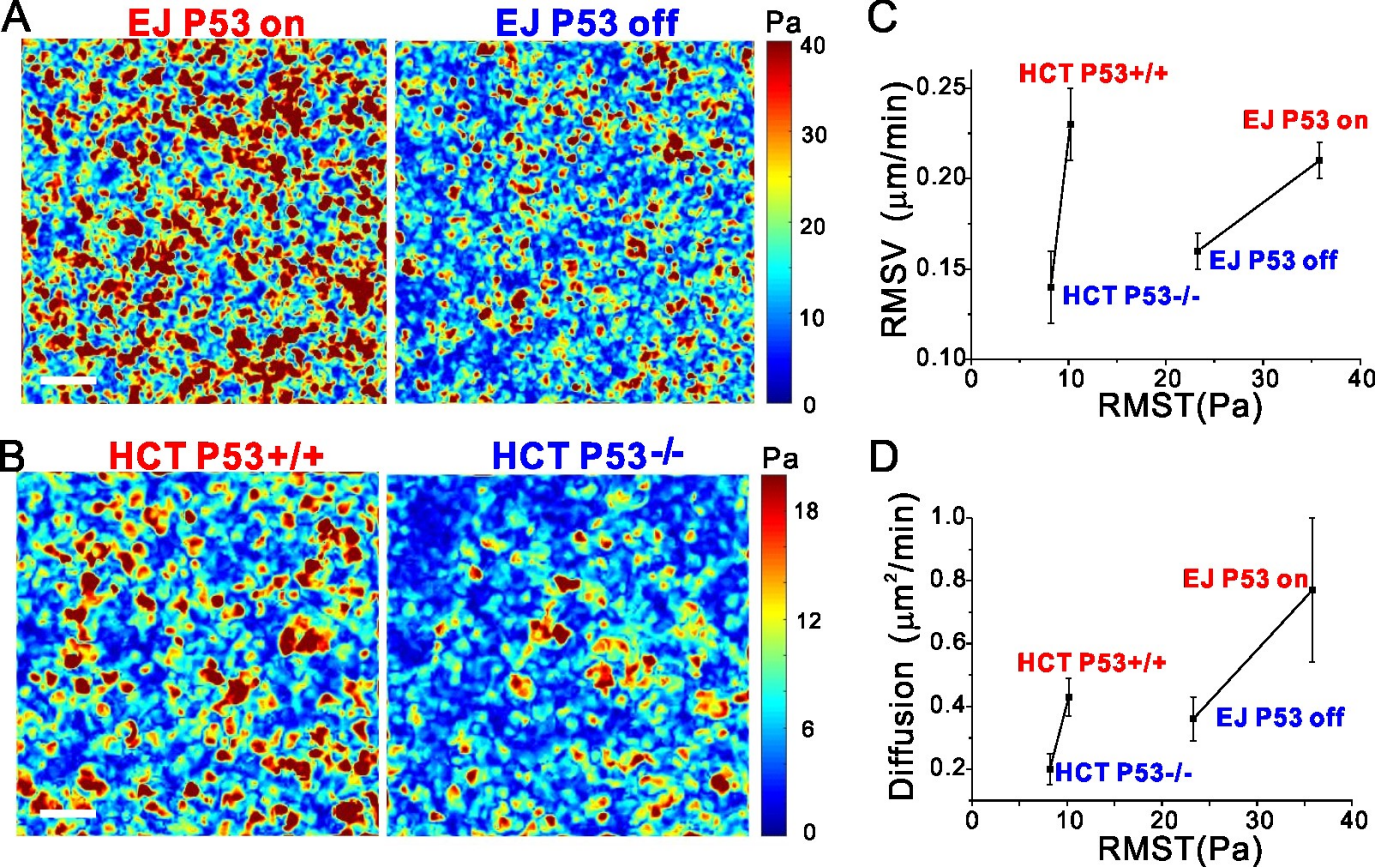
P53 increases traction exerted by the cell layer on the substrate. (**A** and **B**) Representative traction maps from the p53 null cells have fewer hot spots than do p53 expressing cells. (**C** and **D**) In EJ or HCT 116, due to p53 expressing both root mean squared velocities (RMSVs) and diffusion coefficients D increase with increasing RMST. For EJ cells, RMSVs and diffusion coefficients increase to respectively 1.4 times and 2.2 times; for HCT 116 cells, RMSVs and diffusion coefficients increase to respectively 1.6 times and 2.1 times.

Cell-substrate physical interactions play a critical role in collective cellular migration, but to our knowledge the effects of p53 on cell-substrate interactions have not yet been reported. Based on bead displacements, we therefore measured the physical forces exerted by the confluent layer on the gel using Traction Force Microscopy (Methods and Fig 1A). As shown in the representative traction maps (Fig 2A and 2B), p53 null cells had fewer traction hot spots than did p53 expressing cells. Moreover, root mean square tractions (RMSTs) from p53 null cells were smaller than in p53 expressing counterparts; RMSTs were 23.3±0.3 versus 35.9±4.1 Pa, p = 0.01 for EJ; 8.2±0.3 versus 10.2±0.3 Pa, p = 0.1 for HCT 116. Thus expressing p53 caused cells to exert larger traction forces upon their substrates.

With increasing the traction forces in EJ or HCT 116 p53 expressing cells, both the root mean squared velocities (Fig 2C) and diffusion coefficients (Fig 2D) increased. In addition, the more highly diffusive p53 expressing cells did not show longer persistence time of cell migration (S3 Fig). These results, taken together, suggest that those confluent cell layers that express p53 generate larger propulsive traction forces which then accelerate cell motions to promote cell diffusion.

### P53 null cells exhibit more organized cortical actin rings and reduced front-rear cell polarity

**Fig 3:**
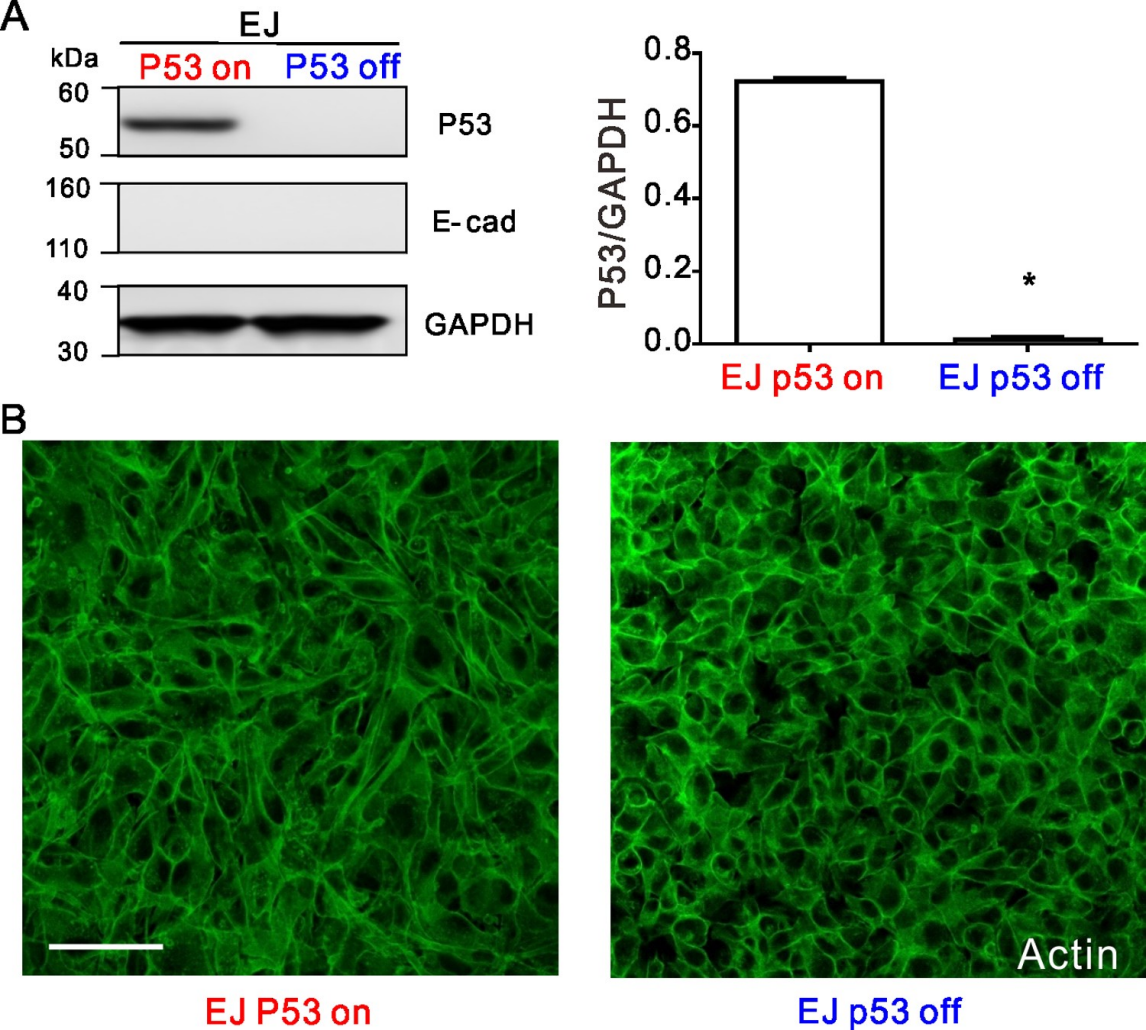
The expression of p53, E-cadherin and actin in the EJ cells. (**A**) Western blot cropped from same gel confirms that the p53 expression in the EJ p53 off cells are null, and E-cad is not detectable. As shown in the histogram p53/GAPDH is 0.71±0.01 versus 0.01±0.01, p<0.0001. The full-length blots are presented in S4A Fig. (**B**) Fluorescent staining shows that actin rings are more organized in the EJ p53 off cells than in EJ p53 on cells. E-cadherin is also not detectable in fluorescent staining (data not shown). Scale bar, 100μm. The fluorescent images represent at least six field views from two experiments. These western blot and fluorescent staining for both EJ and HCT 116 are performed in the same condition (Methods), and as shown in Fig 4 E-cadherin expression in HCT 116 cells serves as the positive control.

**Fig 4:**
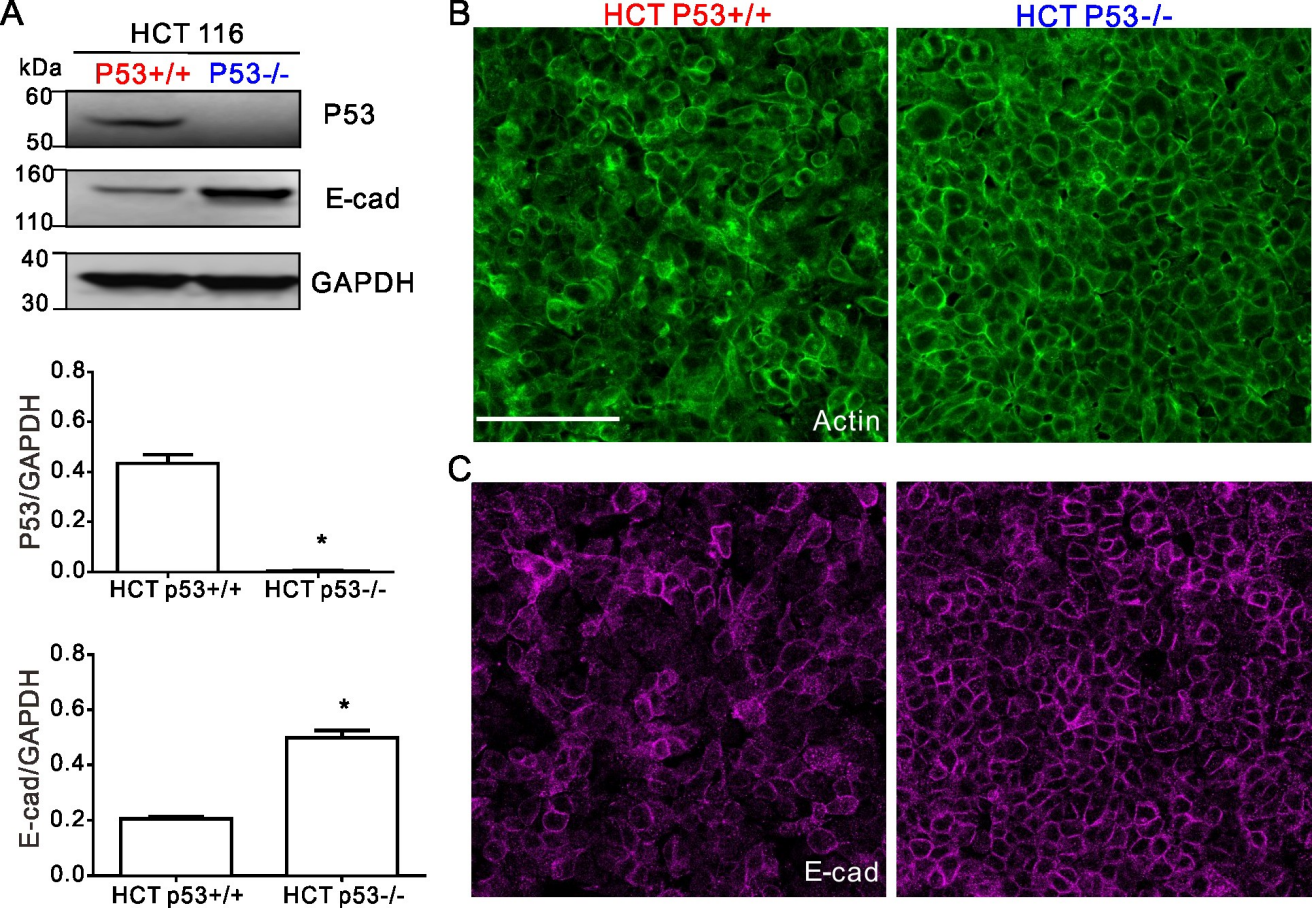
The expression of p53, E-cadherin and actin in the HCT116 cells. (**A**) Western blot cropped from same gel confirms that the p53 expression in HCT 116 p53^-/-^ cells are null. As shown in the histogram p53/GAPDH is 0.43±0.03 versus 0.002±0.004, p<0.0001. E-cadherin expressions in HCT 116 p53^-/-^ cells are ~2.5-fold higher than HCT 116 p53^+/+^ cells (E-cad/GAPDH, 0.50±0.03 versus 0.20±0.01, P<0.0001). The full-length blots are presented in S4B Fig. (**B**) Fluorescent staining shows that actin rings are more organized in the p53^-/-^ cells than in the p53^+/+^ cells. (**C**) Fluorescent staining shows that E-cadherin is located at the cell-cell junction in both the p53^+/+^ and p53^-/-^ cells, and more in the p53^-/-^ cells than the p53^+/+^ cells. Scale bar, 100μm.

In the bladder carcinoma EJ p53 on and EJ p53 off, and in the colorectal carcinoma HCT116 p53^+/+^ and HCT116 p53^-/-^, western blots confirmed expected p53 expression or lack thereof (Figs 3A and 4A). Fluorescent staining with phalloidin showed that the apical cortical F-actin rings found in both of the p53 null carcinomas (EJ p53 off and HCT116 p53^-/-^) were more round and intact than their p53 expressing counterparts (Figs 3B and 4B). However, expression of E-cadherin varied in EJ and HCT 116. In western blotting and fluorescent staining assays, E-cadherin was not detectably expressed in both EJ p53 on and EJ p53 off (Fig 3A, fluorescent staining not shown). Alternatively, E-cadherin was expressed and localized to the cell-cell junctions in both HCT116 p53^+/+^ and HCT116 p53^-/-^, with the latter showing ~2.5-fold greater levels of E-cadherin than the former (Fig 4A and 4C). While a consistent relationship existed between these two different carcinomas, loss of p53 reduced motility and increased cortical actin. No such correlations was found regarding their E-cadherin expression.

### P53 null cells show reduced formation of cryptic lamellipodia

**Fig 5:**
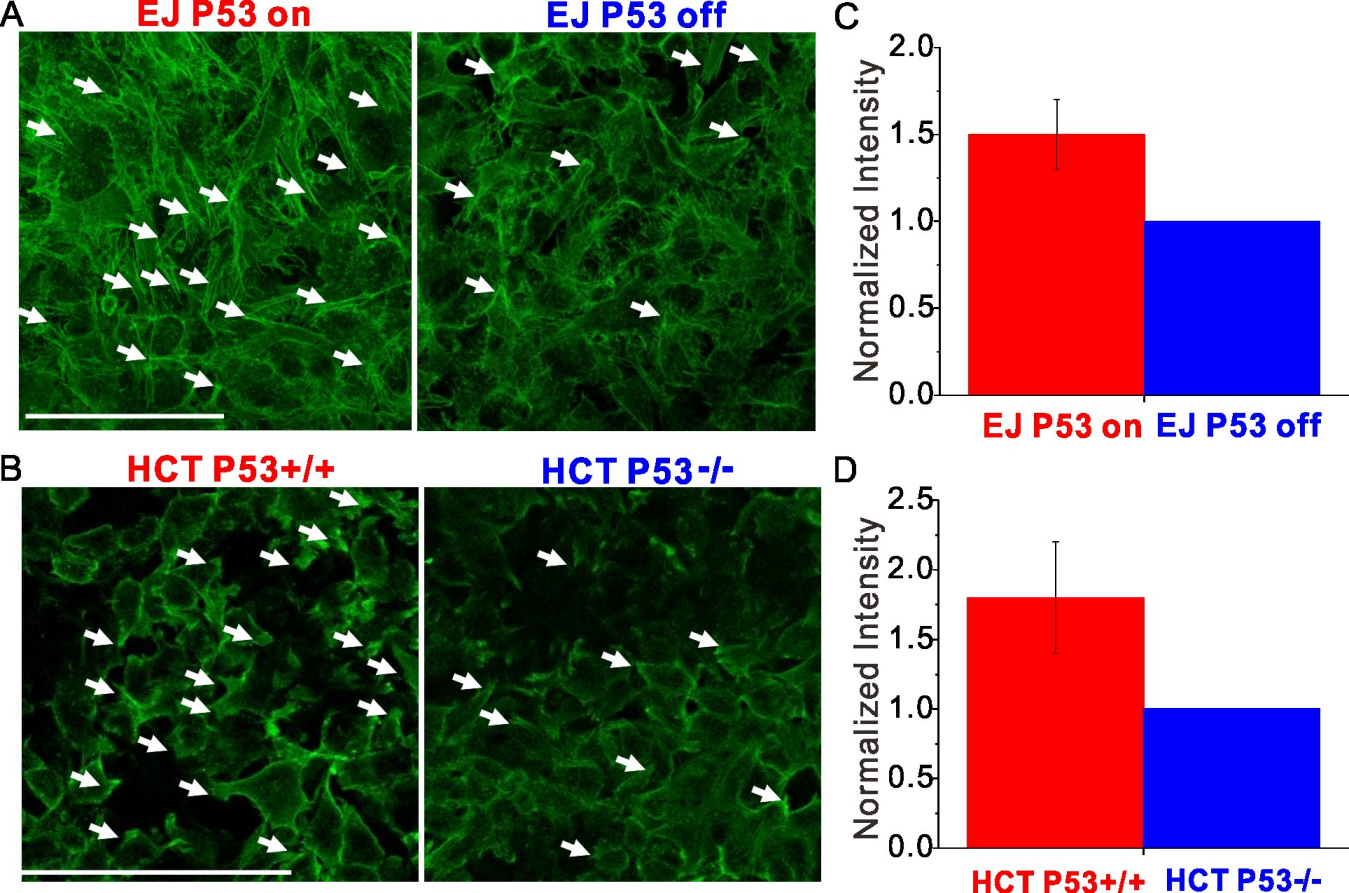
Near basal plane of the cell layers p53 null cell shows reduced appearance of cryptic lamellipodia. (**A** and **B**) Near the basal plane of the cell layer, F-actin tips indicated by the arrows showed the appearance of cryptic lamellipodia. For both EJ and HCT 116, p53 null cells showed reduced appearance of the cryptic lamellipodia. (**C**) F-actin intensity for EJ p53-on cells are 1.5±0.2 times than that for EJ p53-off cells. (**D**) F-actin intensity for HCT 116 p53+/+ cells are 1.8±0.4 times higher than that for HCT 116 p53-/- cells. Scale bar, 100μm.

Fluorescent images of F-actin were obtained using an inverted confocal microscope (Leica SP8, Methods). Near the basal plane of both EJ and HCT 116 carcinomas, the F-actin images showed cryptic lamellipodia (the F-actin tips labeled by arrows in Fig 5A and 5B). We found that loss of p53 was associated with reduced appearance of the cryptic lamellipodia, which are typically associated with collective cellular migration. Moreover, normalized intensity measurements of fluorescent F-actin showed that near the basal plane those cells expressing p53 showed increased F-actin intensity, 1.5±0.2 times for EJ cells, and 1.8±0.4 times for HCT 116 cells (Fig 5C and 5D).

### P53-null multicellular spheroids exhibit lower 3D invasiveness

**Fig 6:**
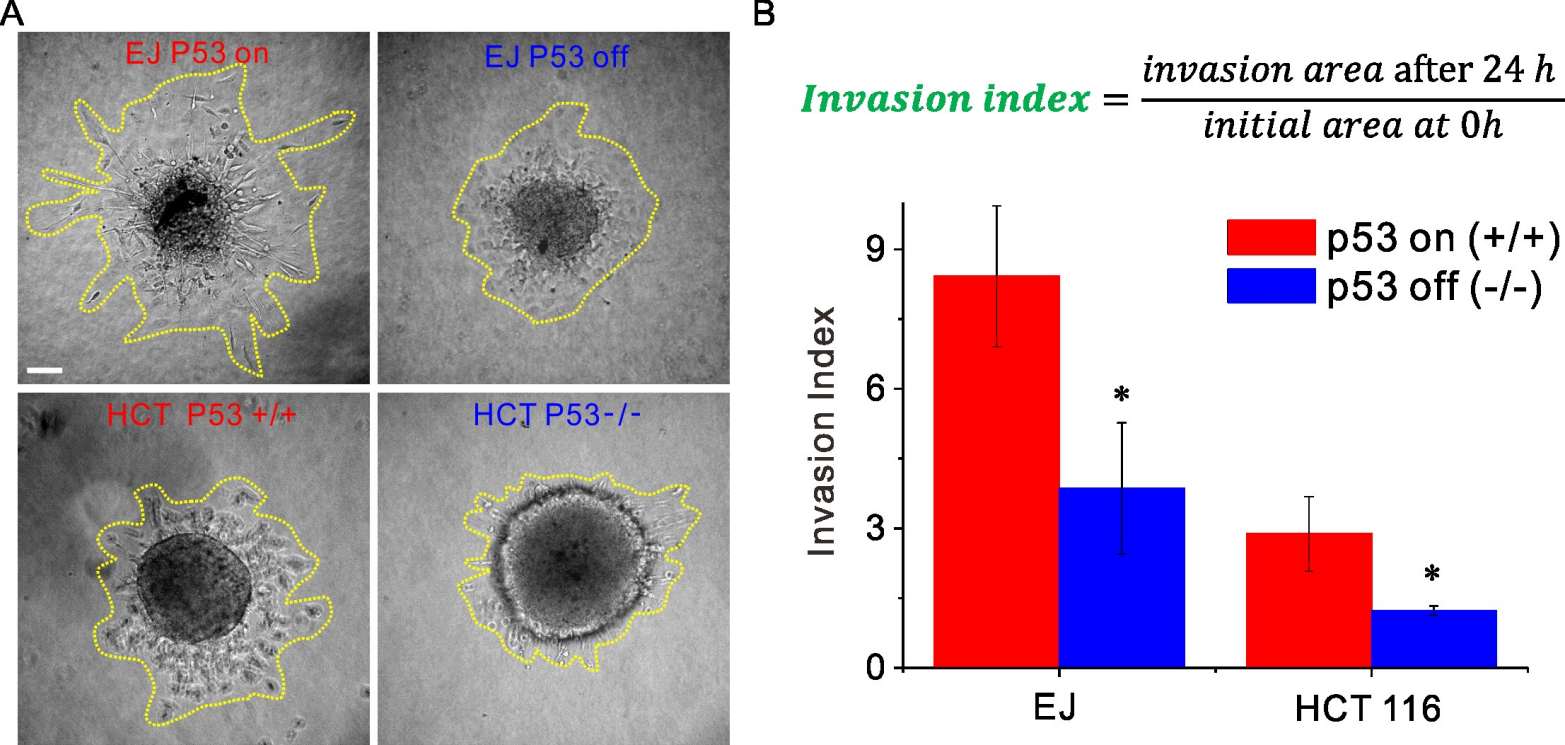
In 3-D collagen matrix, the multicellular spheroids formed by the p53 null cells are less invasive than the ones formed by the p53 expressing counterparts. (**A**) Representative images show the invasion of the multicellular spheroids. The p53 null spheroids invade less than the p53 expressing cells in the both cell lines. The yellow lines indicate the area invaded by cells. (**B**) Invasion index is the area invaded by cells after 24h divided by the initial area. This invasion index for p53 null multicellular spheroids is lower than that from p53 expressing cells (3.8±1.4 versus 8.4±1.5, p<0.0001 for EJ; 1.2±0.1 versus 2.8±0.8, p<0.0001 for HCT 116). Collagen density, 1.5 mg/ml. Scale bar, 100μm.

To better mimic tumor biology, we next conducted studies using 3D multicellular spheroids embedded into collagen matrix. The invasiveness of each carcinoma spheroid was assessed via the invaded area (as enveloped by the yellow dotted line in Fig 6A) normalized by the initial area of the cross section of the spheroid. This metric showed that loss of p53 reduced invasion of the carcinoma spheroids into surrounding matrix (3.8±1.4 versus 8.4±1.5, p<0.0001 for EJ; 1.2±0.1 versus 2.8±0.8, p<0.0001 for HCT 116 in Fig 6B). Compared to their p53 expressing counterparts, p53 null carcinomas (EJ p53 off and HCT 116 p53^-/-^) exhibited less frequent escape of individual cells from the spheroids and less efficient diffusion (S3 and S4 Movies). These results from 3D multicellular spheroids are consistent with those from 2D confluent layer, which both suggest that p53 promotes carcinoma invasion and collective cellular migration.

## Discussion

Using bladder and colorectal carcinoma cell lines in the traditional Boyden chamber assay, here we confirmed that p53 acts to suppress cellular migration [7–9]. However, in these same cell lines in the 2D confluent cell layer, or in the 3D collagen-embedded multicellular spheroid, p53 acts to augment cellular migration. Together, these paradoxical results show that the effects of p53 on cellular migration are context-dependent.

Function of p53 depends on its target genes and proteins, which can exert paradoxical, context-dependent, effects on the same cellular process, including apoptosis, metabolism, differentiation and migration [27]. For example, evidence for regulation of cell migration by p53 is conflicting. The preponderance of the evidence suggests that the tumor suppressor p53 attenuates cell motility and invasiveness through pathways [3–6] such as suppressing the RhoA-Rock or Rac1 [7,8,10] and inhibiting epithelial-mesenchymal transition (EMT) [11,28]. Limited evidence suggests, to the contrary, that p53 promotes *in vitro* and *in vivo* invasion of ovarian carcinoma cells isolated from PTEN; KRas mice [29], and also promotes migration and invasion of human lung, colorectal carcinoma and osteosarcoma cells by activating Rac1 or Rap2a [30,31]. These studies exemplify the paradoxical regulation by p53 in the cell migration from the perspective of different target signal pathways.

### P53 promotes dissemination and invasion of carcinoma cellular collectives

In the cellular collective, results from both bladder and colorectal carcinomas support the notion of the tumor suppressor p53 as a promoter of dissemination and invasion. To manipulate p53, two distinct methods were used in the two human carcinoma cell lines: p53 tet-off system of the bladder carcinoma EJ, and wild-type p53 cells and knock out p53 cells of colorectal carcinoma HCT 116 (Method). To quantify the cell diffusion in the 2-D confluent cell layer, we measured cell speed, mean squared displacement (MSD), and the diffusion coefficient (Fig 1). To our surprise, in both EJ and HCT 116 these metrics from the cells expressing p53 were all higher than those from the p53 null counterparts. As such, the tumor suppressor p53 was associated with faster collective cellular migration and easier escape of trapped cells from their neighbors (S1 and S2 Movies). Both the bladder and colorectal carcinoma spheroids in collagen matrix, which better mimic tumor microenvironment, showed consistent results; the tumor suppressor p53 promotes the carcinoma spheroid to invade a larger area (Fig 6). These results demonstrate that the tumor suppressor p53 promotes carcinoma cell escape from their neighbors and more efficient invasion into matrix.

### P53 increases formation of cryptic lamellipodia and migratory traction forces

Compared to p53 expressing counterparts, the p53 null carcinomas shared the features of having more highly organized rings of cortical F-actin, and more rounded and less polarized cell shape (Figs 3B and 4B). These results are consistent with the general consensus that loss of p53 promotes cellular rounding [7,32]. Moreover recent studies have established cell rounding (i.e., lower aspect ratio) as a key physical mechanism to make cell collectives less diffusive and more jammed by increasing the energy barrier for cells to escape from their neighbors (i.e., exchange neighbors in confluent layers) [22,24,33,34].

Cryptic lamellipodia represent a structure for collective cellular migration [35]. Near the basal planes of both the bladder and colorectal carcinoma cellular collectives, fluorescent images of F-actin showed that loss of p53 was associated with the reduced formation of the cryptic lamellipodia (Fig 5A and 5B), which is consistent with the lower motility of the p53 null cells. These results support the notion that p53 can activate the formation of cryptic lamellipodia to promote cell diffusion and invasion in the cellular collectives.

To our knowledge these studies are the first to quantify the mechanical effects of p53 on cell-substrate traction forces. Measurements in both EJ and HCT 116 suggest that the loss of p53 consistently causes these carcinoma cells to exert smaller traction forces on their substrate (Fig 2). It remains unclear, however, if reduction in traction forces might be attributable to reduced lamellipodia formation and less motility [36,37].

### E-cadherin expression

Many studies suggest that p53 can prevent epithelial-mesenchymal transition (EMT) and increase E-cadherin expression to decrease cancer cell motility [11,28,38,39]. Nevertheless, at least one study [9] suggests that loss of p53 does not decrease the E-cadherin expression. Our current studies in the 2-D confluent cell layers also show a discordant relationship between p53 and E-cadherin expression. The bladder carcinomas do not express E-cadherin regardless of expressing p53 or not, which suggests that in this context the effects of p53 on the motility of collective carcinomas are not mechanistically related to E-cadherin expression. Nevertheless, in the bladder carcinoma collective it remains unclear whether the effects of p53 on cell migration are associated with the epithelial-to-mesenchymal transition (EMT), and whether p53 increases expression of other cadherins, such as P- and N-cadherin. For the colorectal carcinoma cells, loss of p53 increases E-cadherin expression, which might contribute to increased cell-cell interaction so as to cage cells by their neighbors, although theoretical models suggest, to the contrary, that increasing cell-cell adhesion decreases the energy barrier for cells to escape from their neighbors [33]. These results suggest that regulation of E-cadherin expression by p53 is context-dependent, and may differ for different carcinoma types.

### Microenvironment, geometry and collectivity

Data show that p53 augments cell migration in both the 3D multicellular spheroid and in the 2D confluent cell sheet but suppresses cell migration in the pore of the Boyden chamber. It is clear that each of these three assays imposes upon the migrating cell strikingly different geometrical constraints. For example, each cell within the core of the spheroid is fully surrounded in 3D by neighboring cells, and the potential for cell-cell interactions is therefore unconstrained by any fixed system boundary. By contrast, each cell within the planar cell sheet is constrained from above by fluid medium and from below by a fixed planar substrate. And each cell in the pore of a Boyden chamber is constrained over most of its surface by the fixed tubular boundary of the pore. As regards geometrical constraints imposed by fixed system boundaries, these three assays therefore present the migrating cell with a spectrum of possibilities, each permissive of a different degree of cell-cell contact and, as a result, a different degree of cell-cell interaction, cooperativity, and collectivity. It is within the narrow pore of the Boyden chamber that the effects of cell-cell interaction, cooperativity, and collectivity are reasoned to the most constrained, and it is in that case alone that p53 is seen to suppress cellular migration rather that augment it.

## Conclusion

The tumor suppressor p53 attenuates cellular migration in the Boyden chamber assay, but paradoxically promotes cellular migration in the confluent 2-D cell layer and the 3-D spheroidal cell cluster. Compared to p53 expressing cell layers, p53 null cell layers show lower tractions, higher cell-cell adhesion, and less polarized morphology (S5 Fig). Importantly, whether restoration of p53 function in p53-null cells could reverse these phenotypic changes remains to be studied. Together, these paradoxical results show that the effects of p53 on cellular migration are context-dependent, and suggest, further, that wild type p53 might have the potential to promote carcinoma migration rather than suppress it.

## Methods

### Cell culture

Human bladder carcinoma EJ cells [40] were cultured in Dulbecco’s modified Eagle’s medium (DMEM, Corning, 10-013-CV) containing 10% Fetal Bovine Serum (FBS, Atlanta Biologics), 100 μg/ml of Hygromycin (Sigma, H3274), and 200 μg/ml Geneticin G418 (Teknova, G5005). P53 knock-out EJ cells (EJ p53 off) and p53 expressing EJ cells (EJ p53 on) were established respectively by the presence and the absence of 1 μg/ml doxycycline (Sigma, D9891) in the culture media. Human colorectal carcinoma HCT116 cells [41] were cultured in DMEM containing 10% FBS. Both EJ and HCT 116 cells were maintained at 5% CO_2_ and 37°C. All the human cell lines and experimental protocols were approved by Harvard Institute Review Board and carried out in accordance with the relevant guidelines and regulations. The datasets generated during the current study are available from the corresponding author on reasonable request.

### Single cell invasion assay

The 24-well Boyden chamber (Corning, 354480) contains an 8 μm pore size PET membrane which has been coated by Matrigel. We added warmed serum-free culture medium into the interiors of the chambers and the bottoms of the wells, and allowed the system to rehydrate for 2 hours in humidified tissue culture incubator. After rehydration, we carefully removed the medium without disturbing the layer of Matrigel. We prepared the serum-free cell suspension (5×10^4^ cells/ml for EJ, 2×10^5^ cells/ml for HCT 116), and then added 0.5ml into the chamber. We used sterile forceps to transfer the chambers to the wells containing 0.75ml culture medium containing serum as chemoattractant. Cells were incubated in the chambers for 22 hours in 37°C, 5% CO_2_ incubator. After the incubation, we used cotton tipped swabs to scrub the surface of the chamber twice to remove the non-invading cells from the upper surface. Cells were then fixed and stained for F-actin and nucleus via the above methods. We counted the nuclei of the entire well bottom through the particle analysis in Image-J. N = 4 for each type from two experiments.

### 2-D confluent cell layer assay

Polyacrylamide gel substrates (Young’s modulus, 1.2kPa, thickness, 100μm) were fabricated on dishes, and for the traction measurement 0.5μm fluorescent red beads (Invitrogen, F8823) were embedded near the gel surface [21]. On the gel we then coated an 8×8mm^2^ square region with 0.1 mg/ml collagen (Advanced Biomatrix, 5005). 80 μl cell suspension (5×10^5^ cells/ml for EJ, and 8×10^5^ cells/ml for HCT 116) were seeded on the collagen coated region. After 24h, both phase contrast images and fluorescent images were captured for the cells and the beads respectively via an inverted fluorescent microscope (Leica DMI8) (10 min interval for 48 h at 5% CO_2_ and 37°C). The phase images were used to quantify cell motion via our custom-written software based on the function in MATLAB termed as opticalFlowFarneback, and we reported the cell velocity map and the mean squared displacement (MSD) as shown in Fig 1. The fluorescent images of the red beads were used to calculate the gel deformation, as described in following.

### Traction force microscopy

Traction was quantified by traction force microscopy (TFM) [42]. At the end of the 2-D confluent cell layer assay, cells were detached with trypsin (Corning, 25-052-CI), then we captured the fluorescent images of the red beads as the reference for the gel deformation. The gel deformation was calculated via our custom-written particle image velocimetry software. Based on the gel deformation field, the traction was computed via constrained Fourier transform traction [42]. We reported the traction map and root mean squared traction (RMST) in Figs 4 and 5.

### 3-D multicellular spheroid assay

A 200 μl cell suspension (2×10^5^ cells/ml for EJ p53 on, 5×10^5^ cells/ml for HCT 116) was cultured in an ultra-low attachment 96-well plate (VWR, 29443–034) to form the multicellular spheroid. After 48h, each spheroid was carefully pipetted into 1.5mg/ml collagen matrix (Advanced Biomatrix, 5005). Cell culture medium was used to adjust the collagen concentration, and the collagen matrix was equilibrated through 10X PBS (volume ratio, 1:10 between 10X PBS and collagen) and 1M NaOH (0.5% of total matrix volume). These processes were performed on ice to avoid collagen polymerization. We then moved the collagen matrix into 37°C incubator to induce collagen polymerization. After 1h, we monitored the invasion of the spheroids via Leica microscope (Leica DMI8).

### Immunofluorescence and confocal laser-scanning microscopy

For immunofluorescence analysis of cell lines, cell layers from the 2-D assays were fixed in 4% paraformaldehyde/PBS for 10 min, permeabilized and blocked in 0.2% Triton X-100/PBS containing milk for 20 min. The cell layers were stained with primary E-cadherin antibody (Invitrogen, 334000) [11], then stained with secondary antibodies Alexa Fluor 488 (Invitrogen, A-11029). F-actin was stained with Phalloidin conjugated with Alexa Flour 594 (Invitrogen, A12381). LSM (laser scanning microscopy) images were captured by using inverted confocal microscope (Leica SP8, 40x/0.8 oil objective). These images were then processed by using same setting in Image J. All the florescent images represented at least six field views from two experiments.

### Western blot

Protein expression was determined by western blot. All experiments were performed at 72 hours to ensure adequate protein expression. Cells were washed twice with cold PBS and then cell lysates were collected on ice with 10μg phosphatase inhibitor cocktail (Roche). Equal amount of protein lysates from each condition were separated by using NuPAGE 15 well 4–12% Bis-Tris protein gel (Thermo Fisher, MA), then transferred onto nitrocellulose membrane. The membrane was cut into three and probed separately with E-cad antibody (Invitrogen, 334000), p53 antibody (cell signaling, 2527S) and GAPDH antibody (GeneTex, 627408), followed by secondary antibody (Abcam, 205718 & 97040). Here we used GAPDH as our loading control, because the variation of GAPDH expression among different donors/transfections was less than 1 fold (data not shown here). These antibodies have been confirmed by manufacture and previous studies [11,43].

## Supporting information

S1 Fig P53 inhibits invasion of the carcinoma cell in Boyden chamber. (**A**) shows the representative images for the bottom of the Boyden chamber (green for F-actin, blue for nucleus). The p53 null cells invade more than the p53 expressing counterparts. (**B**) The numbers of the cells per well from the p53 null cells are higher than those from p53 expressing counterparts, (125.0±52.7 versus 415.2±100.7 for EJ; 248.8±64.6 versus 891±238.8 for HCT 116). Scale bar, 200μm.
(TIF)

S2 Fig Bright Field images of the Boyden chamber top surface with pores of 8 μm diameter after the assay. After finishing the 22h assay, Most of cells on the chamber top surface are still reasonably separated. Scale bar, 100μm.
(TIF)

S3 Fig Persistence time of cell migration is assessed by using the persistence time τ = 4D/v^2^ in which D is the diffusion coefficient and v the RMSV. For EJ the averaged persistence time of p53 expressing cells is 1.2 times higher than p53 null, but there is no significant difference (p = 0.7). For HCT 116, however, the averaged persistence time of p53 wild type cells is 0.8 times lower than the p53 null (p = 0.01). Scale bar, 100μm.
(TIF)

S4 Fig Western blot of p53, E-cadherin and GAPDH for EJ and HCT 116. Exposure time is 10s for GAPDH, 60s for E-cadherin for both EJ and HCT 116 cells, and 10s and 30s for p53 of EJ cells and HCT 116 cells respectively.
(TIF)

S5 Fig Illustration for the differences between the p53 null and p53 expressing collective cells. Compared to p53 expressers, p53 null cells exhibit more organized cortical actin rings together with reduced front-rear cell polarity and less formation of cryptic lamellipodia. Moreover our study show that p53 increases the traction exerted by the collective cells on substrate, and promotes diffusion and invasion of the collective cells.
(TIF)

S1 Movie Cell migration in the 2-D confluent EJ cell layer. (AVI)

S2 Movie Cell migration in the 2-D confluent HCT 116 cell layer. (AVI)

S3 Movie Cell invasion of the 3-D EJ spheroid. (AVI)

S4 Movie Cell invasion of the 3-D HCT 116 spheroid. (AVI)
