# Supplementary figures and images for: The tumor suppressor p53 can promote collective cellular migration
Source: PLoS One. 2019 Feb 1;14(2):e0202065. doi: 10.1371/journal.pone.0202065 (PMC6358060; doi:10.1371/journal.pone.0202065)

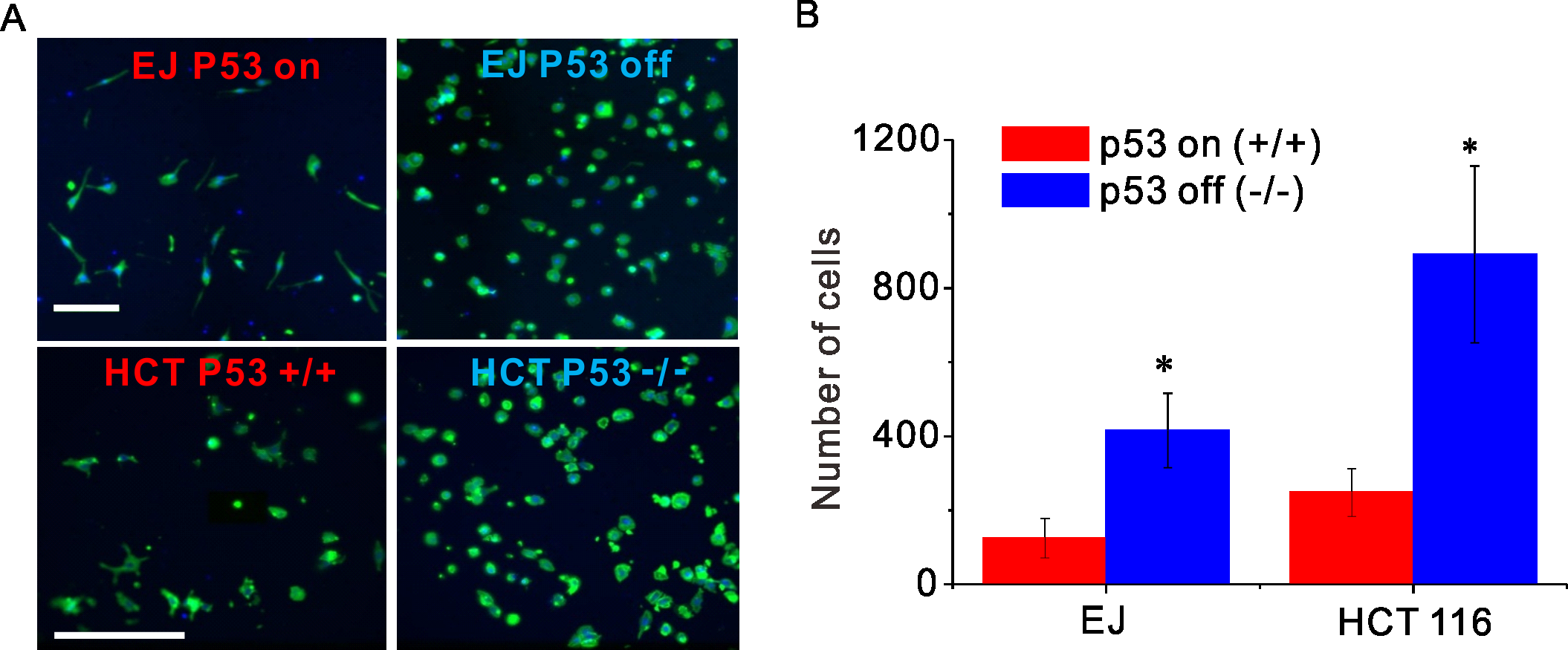

Supplement: S1 Fig — (A) shows the representative images for the bottom of the Boyden chamber (green for F-actin, blue for nucleus). The p53 null cells invade more than the p53 expressing counterparts. (B) The numbers of the cells per well from the p53 null cells are higher than those from p53 expressing counterparts, (125.0±52.7 versus 415.2±100.7 for EJ; 248.8±64.6 versus 891±238.8 for HCT 116). Scale bar, 200μm. (TIF) [file pone.0202065.s001.tif]

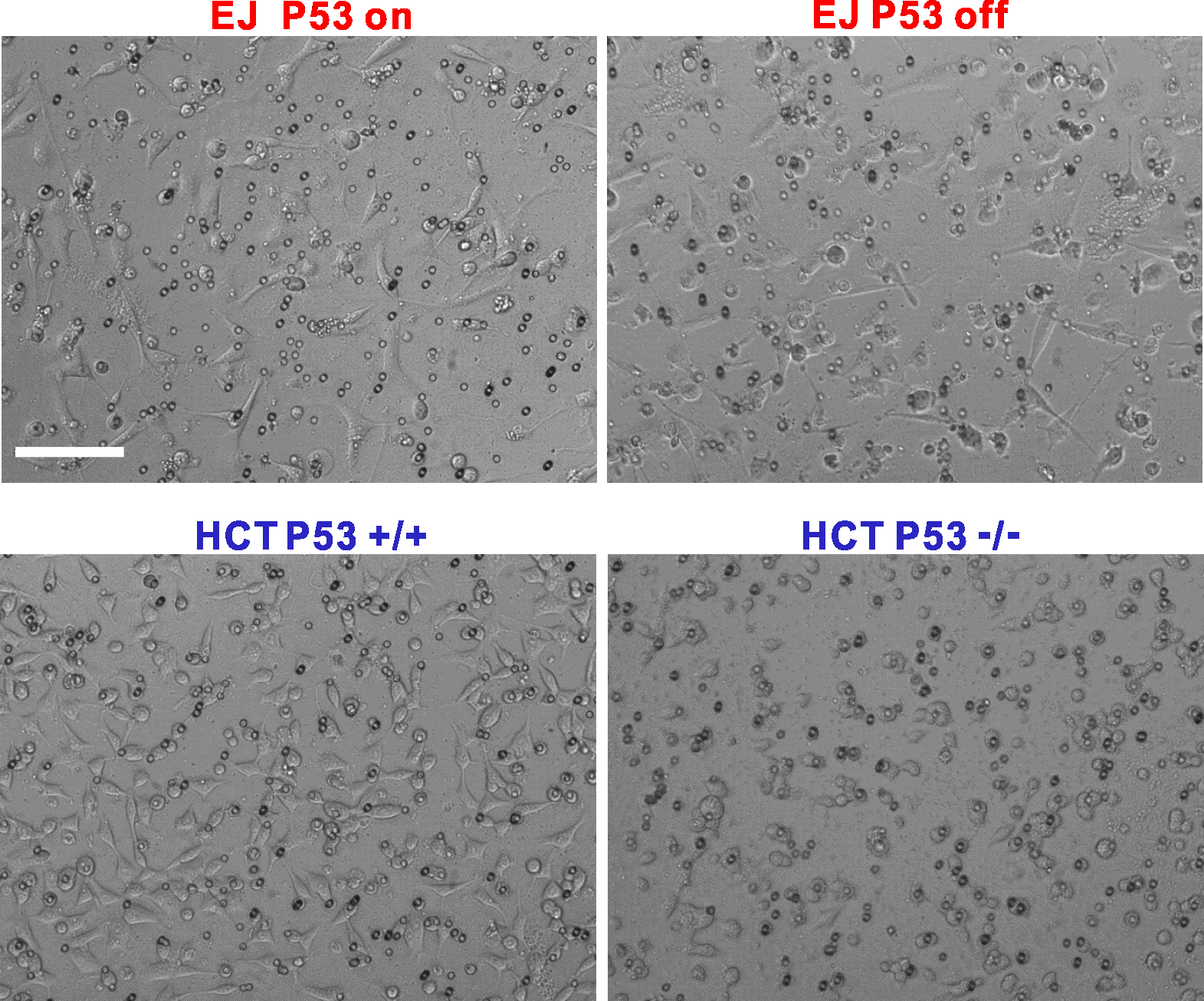

Supplement: S2 Fig — After finishing the 22h assay, Most of cells on the chamber top surface are still reasonably separated. Scale bar, 100μm. (TIF) [file pone.0202065.s002.tif]

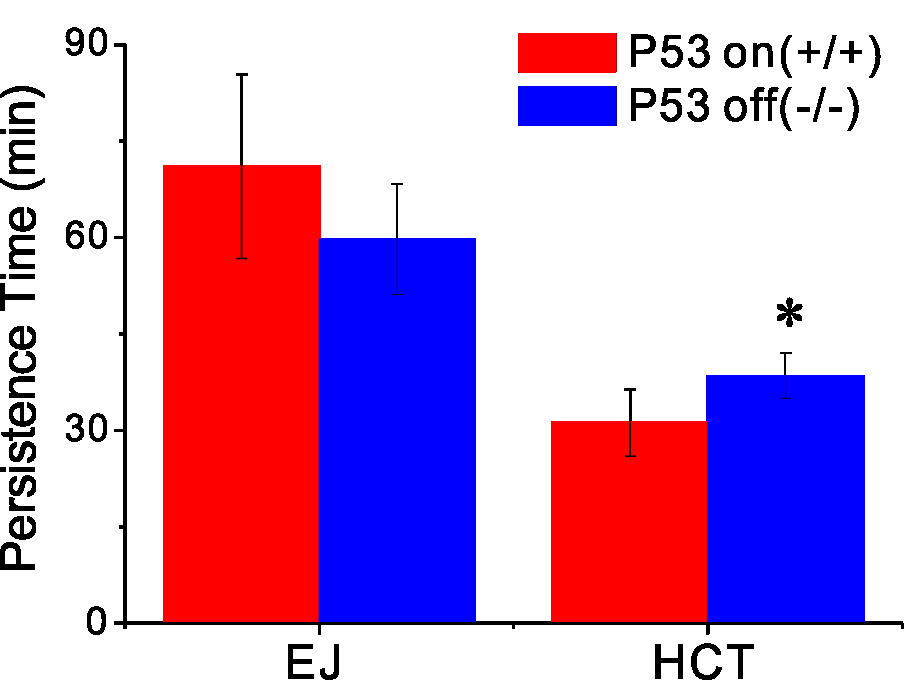

Supplement: S3 Fig — For EJ the averaged persistence time of p53 expressing cells is 1.2 times higher than p53 null, but there is no significant difference (p = 0.7). For HCT 116, however, the averaged persistence time of p53 wild type cells is 0.8 times lower than the p53 null (p = 0.01). Scale bar, 100μm. (TIF) [file pone.0202065.s003.tif]

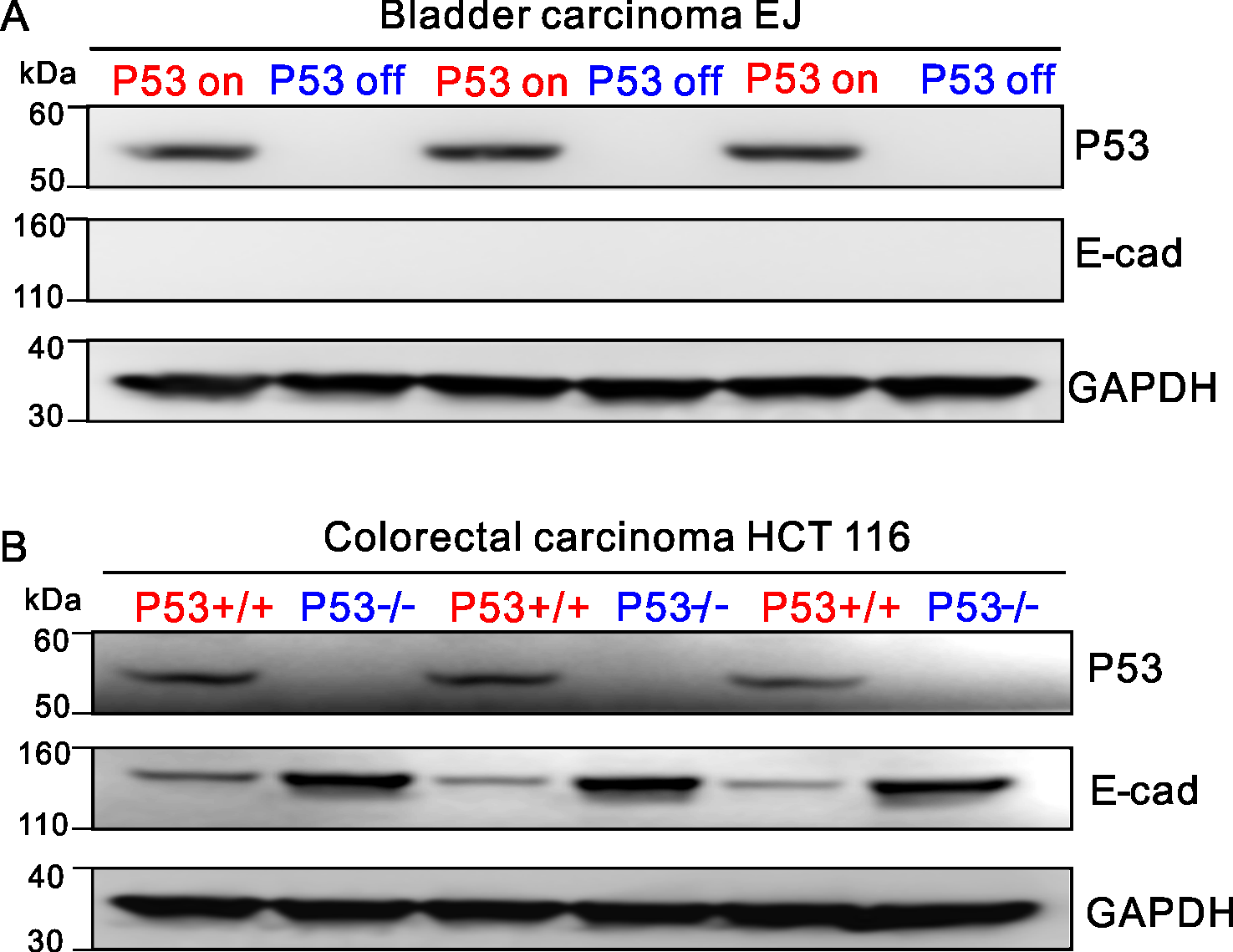

Supplement: S4 Fig — Exposure time is 10s for GAPDH, 60s for E-cadherin for both EJ and HCT 116 cells, and 10s and 30s for p53 of EJ cells and HCT 116 cells respectively. (TIF) [file pone.0202065.s004.tif]

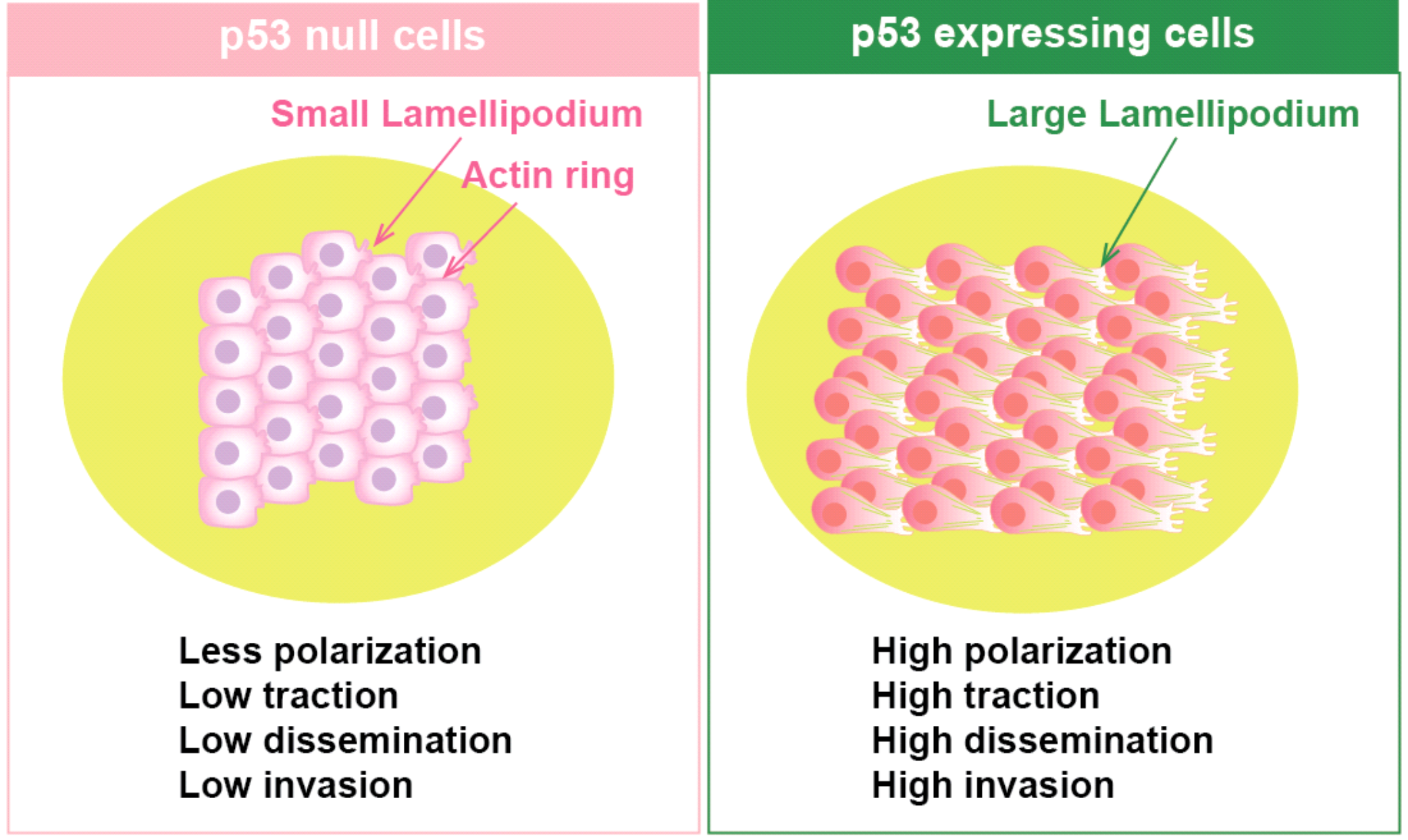

Supplement: S5 Fig — Compared to p53 expressers, p53 null cells exhibit more organized cortical actin rings together with reduced front-rear cell polarity and less formation of cryptic lamellipodia. Moreover our study show that p53 increases the traction exerted by the collective cells on substrate, and promotes diffusion and invasion of the collective cells. (TIF) [file pone.0202065.s005.tif]
